# Congenital scoliosis – Quo vadis?

**DOI:** 10.4103/0019-5413.61997

**Published:** 2010

**Authors:** Ujjwal K Debnath, Vivek Goel, Nanjanduppa Harshavardhana, John K Webb

**Affiliations:** The Centre for Spinal Studies & Surgery, Queens Medical Centre, University Hospital, Nottingham, NG7 2UH, UK

**Keywords:** Congenital scoliosis, hemivertebrae, scoliosis

## Abstract

Congenital spinal vertebral anomalies can present as *scoliosis* or *kyphosis* or both. The worldwide prevalence of the vertebral anomalies is 0.5-1 per 1000 live births. Vertebral anomalies can range from hemi vertebrae (HV) which may be single or multiple, vertebral bar with or without HV, block vertebrae, wedge shaped or butterfly vertebrae. Seventy per cent of congenital vertebral anomalies result in progressive deformities. The risk factors for progression include: type of defect, site of defect (junctional regions) and patient's age at the time of diagnosis. The key to success in managing these spinal deformities is early diagnosis and anticipation of progression. One must intervene surgically to halt the progression of deformity and prevent further complications associated with progressive deformity. Planning for surgery includes a preoperative MRI scan to rule out spinal anomalies such as diastematomyelia. The goals of surgical treatment for congenital spinal deformity are to achieve a straight growing spine, a normal standing sagittal profile, and a short fusion segment. The options of surgery include *in situ* fusion, convex hemi epiphysiodesis and hemi vertebra excision. These basic surgical procedures can be combined with curve correction, instrumentation and short segment fusion. Most surgeons prefer posterior (only) surgery for uncomplicated HV excision and short segment fusion. These surgical procedures can be performed through posterior, anterior or combined approaches. The advocates of combined approaches suggest greater deformity correction possibilities with reduced incidence of pseudoarthrosis and minimize crankshaft phenomenon. We recommend posterior surgery for curves involving only an element of *kyphosis* or modest deformity, whereas combined anterior and posterior approach is indicated for large or *lordotic* deformities. In the last decade, the use of growing rods and vertebral expandable prosthetic titanium rib has improved the armamentarium of the spinal surgeon in dealing with certain difficult congenital spinal deformities. The goal of growing rod treatment is to provide simultaneous deformity correction and allow for continued spinal growth. Once maximal spinal growth has been achieved, definitive fusion and instrumentation is performed.

## INTRODUCTION

Congenital spinal vertebral anomalies can present as *scoliosis* or *kyphosis* or both. *Congenital scoliosis* is a lateral curvature of the spine that is due to the presence of vertebral anomalies causing an imbalance in the longitudinal growth of the spine. Most *congenital scoliosis* is often recognized at birth, but more subtle spinal defects can remain undetected. A key feature of *congenital scoliosis* is the presence of one or more abnormally formed vertebrae. When these anomalies are identified, the curve should be classified as congenital, even if the deformity is not apparent until adolescence. The worldwide prevalence of the vertebral anomalies is 0.5-1 per 1000 live births.^1^

## EMBRYOLOGY

The spine is formed during a process called somitogenesis. This formation takes place between the third and fourth week of gestation. In this process, segments of mesodermal tissue called somites are formed in pairs surrounding the eventual spinal cord. The antero-medial wall of the somite is called sclerotome. These somites are regularly sized and spaced, and this careful organization is essential for the normal patterning of the spine. These same somites also form the axial muscles that connect the vertebral segments, and the ribs associated with the *thoracic* vertebrae. Cells from the sclerotome spread out centrally to form an unsegmented, cellular perichondral sheath, which eventually forms the vertebral body. In the notochord, alternating zones of loose and dense zones appear (superior zonal cells forms the centrum of vertebra and the inferior zonal cells forms the intervertebral disc). Many other organs and tissues are being made during this important time in development, including the heart, kidneys, brain, limbs and other organs. Complex cellular cytoskeletal rearrangements and biomechanical changes occur during the segmentation phase.^2^ Congenital vertebral defects identical to those in *congenital scoliosis* have resulted from disruption in somitogenesis as evident from animal models. Multiple theories have been suggested to explain the congenital vertebral anomalies e.g. failure to ossify, osseous metaplasia of annulus fibrosus or persistent notochord.^3^

## GENETICS

A close interaction of genes and environment regulates the development of normal spine. Developmental studies in animal models have identified many genes regulating somite formation and segmentation. Recently, genes in the “notch” family have been shown to regulate development of vertebral precursors in the mouse^4^–^8^ and defects in human notch genes have been associated with congenital vertebral defects.^9^ Other genes which have been shown to be associated with *congenital scoliosis* are Pax1, DLL3 etc.^10^,^11^ Environmental factors also affect the delivery of the genetic instructions during development e.g. maternal diabetes and ingestion of anti-epileptic drugs during pregnancy.^12^

Wynne–Davies (1975) suggested that there is 1 in 100 risk of a first degree relative having a single vertebral malformation and a risk of 1 in 10 for multiple vertebral anomalies in either siblings or children of a patient.^13^

## CLASSIFICATION

The classification of *congenital scoliosis* is based upon the embryologic maldevelopment of the spine. The Scoliosis Research Society has accepted the classification of spinal deformities proposed by Goldstein and Waugh (1973) which includes a classification of congenital deformities devised by MacEwan *et al*. (1968) with subdivision into *scoliosis, kyphosis or lordosis*.^14^,^15^ The embryological etiology may be a failure of bone formation, a failure of bone segmentation, or both. Failures of formation may be complete and unilateral hemivertebrae (HV) or partial and unilateral (wedge vertebrae). Failures of segmentation may be unilateral, producing “bars,” or bilateral, producing “blocks”.

## MORPHOLOGY

The absence of one pedicle and one half of the vertebral body results in HV. It may be unsegmented when it is fused with the adjacent vertebral bodies; partially segmented when fused to the vertebral body either above or below; a fully segmented one which is separated from the body above and below by a disc space. HV can occur in the ipsilateral adjacent levels of spine producing asymmetrical growth. HV may be counterbalanced by another one on the contra lateral side separated by one or several healthy vertebrae called hemimetameric shift.^16^ HV usually occurs as extra spinal segments and is often accompanied by an extra rib. They may result from an abnormal cleavage of the primary ossification center.

The unilateral bars may act as growth tether and sometimes may span an ipsilateral formation defect, resulting in a unilateral bar and a contra lateral HV.^17^ The mixed type may be difficult to define at birth since only 30% of spine is ossified. Most malformations occur at the apex of the curve. Most common curvatures are *thoracic* (64%) followed by *thoracolumbar* (20%), *lumbar* (11%) and *lumbosacral* (5%).^18^

## ASSOCIATED ANOMALIES

Patients with *congenital scoliosis* frequently have other associated anomalies. Sometimes rib fusion or absence can be observed along with the spinal anomalies since ribs are formed in close association with the vertebrae. When the number of ribs on the right or left side do not match, congenital vertebral anomalies should be suspected. Associated congenital anomalies can also involve non-skeletal organs.^19^ About 20 to 40% of patients with congenital vertebral malformations have renal anomalies, including unilateral kidney, ureteric duplication or obstruction.^20^,^21^ All patients with *congenital scoliosis* should have mandatory renal ultrasound or magnetic resonance imaging (MRI) scans. Opitz (1982) described the developmental field concept which gives an insight into the understanding of multisystem involvement; a defect in one system should direct us to the evaluation of others.^22^

A second area of concern is the detection of cardiac anomalies. About 10-15% of patients with *congenital scoliosis* have congenital heart defects ranging from atrial and ventricular septal defects to tetrology of fallot or transposition of great vessels.^23^ A careful cardiovascular system evaluation with a screening echocardiogram is essential for these children. Restricted pulmonary function in patients with severe curves especially beyond 90° is of major concern since there is evidence of hypoplastic lung development.^24^ All patients require respiratory function tests, especially vital capacity screening. Most organ defects are observed in the mixed defects (73%) followed by failure of formation (47%) and failure of segmentation (37%).^21^

Patients with segmented or mixed defects are at higher risk (35%) of having a neural axis abnormality e.g. diastematomyelia (split cord), tethered cord, Chiari malformation and intradural lipomas.^3^ Up to 20% patients with *congenital scoliosis* have diastematomyelia, which should be addressed by resection prior to correcting the spinal deformity. Similar surgical treatment is required for other intraspinal anomalies before addressing the congenital spinal curvature. Routine MRI scan is essential in the evaluation of these patients.^25^ The coronal and sagittal images may help to define the segmentation and formation defects in the anterior spine. The axial views can be viewed to look at the pedicle anatomy of the patient when contemplating transpedicular instrumentation.

Congenital scoliosis is associated with syndromes; Goldenhaar and VACTERL being the commonest associations followed by Klippel-Feil, Alagille, Jarcho Levin, Joubert, basal cell naevus, trisomy^18^ and diabetic embryopathy.^10^ Musculoskeletal anomalies associated with vertebral malformation are clubfeet, Sprengel's deformity, developmental dysplasia of hip and skeletal dysplasias.

## NATURAL HISTORY

Congenital curves tend to be very rigid and resistant to correction. The natural history of the individual curve should be understood, and deformities with relentless progression should not be allowed to worsen. In general, 25% congenital curves don't progress, 50% curves progress slowly and 25% progress rapidly. In those with a known tendency for progression, early surgical intervention, such as spinal fusion, is essential and preferable to prevent severe curves to develop. Early surgical intervention for children with congenital deformities that have a poor prognosis allows for additional growth in the involved areas of the spine.

The formation and segmentation defects usually have serious consequences in spinal growth during childhood. The severity of the congenital deformity depends on the type of anomaly, the site of occurrence, and the overall growth potential of the individual.^26^ Winter *et al*. (1968) and later McMaster *et al*. (1982) reported that the rate of deterioration and the severity of spinal deformity can be predicted by assessing the type of anomaly and curve location.^26^,^27^ Approximately 75% patients required surgical fusion and 84% who were left untreated developed curves greater than 40° at skeletal maturity. *Thoracolumbar curves* tend to be more severe than other locations. Children with clinical deformities in the first year of life had the worst prognosis with early progression of curve magnitude. Generally curve progression occurs more rapidly during the first five years of life and again during the adolescent growth period.^28^

In the normal spine, growth occurs symmetrically at the endplates on the upper and lower end of vertebrae leading to balanced spine in both coronal and sagittal plane. In the presence of congenital vertebral anomaly, there is imbalance of growth plates on either side of spine resulting in localized unilateral longitudinal growth imbalance and leading to increase in spinal deformity as child grows. Growth potential may also be inferred from assessment of the surrounding discs. Fully segmented vertebrae with healthy definable discs above and below have much more potential to cause a deformity compared to an unsegmented HV. The presence of a bar or fused ribs is a good predictor of curve progression. Either can act as a tether, and the combination tends to produce even more rapid curve progression. The severity of deformity can be graded from worse to good prognosis: unilateral unsegmented bar combined with single or multiple convex HV, followed by a unilateral unsegmented bar, double convex HV, and a single convex HV, with the block vertebra having the best prognosis.^29^ An unilateral unsegmented bar adjacent to a contra lateral HV is the worst culprit which progresses more than 10° per annum in the *thoracolumbar* region. Sometimes a hemimetameric shift produces a progressive deformity in *thoraco-lumbar* and *lumbosacral* junction.^29^

The curves can be mild to severe in the *cervico thoracic* region. Some curves due to single or multiple HV at the *cervico-thoracic* junction can lead to progressive deformity which would require surgical fusion at an early age.^27^ In the *thoracic* region, an unsegmented unilateral bar is often associated with fused ribs close to the apex of the curve on the concave side. In patients who have moderate or severe angulation, it is important to correct the angulation within this area before spine fusion.^17^,^27^ There is a global loss of trunk height and width leading to restriction in pulmonary function.

In the *thoracolumbar* area, congenital curves in general have the same prognosis as do curves involving the *thoracic* spine. HV which may be located laterally, posterolaterally, or directly posteriorly are often seen. Worse is the *kyphosis* and prognosis as the HV position is more posteriorly located in the spine. In the *lumbar* area, congenital curves may be associated with anomalies in the lower extremities or in the genitourinary system. A curve in this region of as much as 70° may produce little cosmetic deformity if the curve is not decompensated.^27^ Majority of curves in the *thoracic* and *lumbar* region caused by a single fully segmented HV progresses slowly at the rate of 1°-2° per year. The most malignant and deforming type of HV occur at the *lumbo-sacral* junction which needs urgent attention.

**Figure 1:**
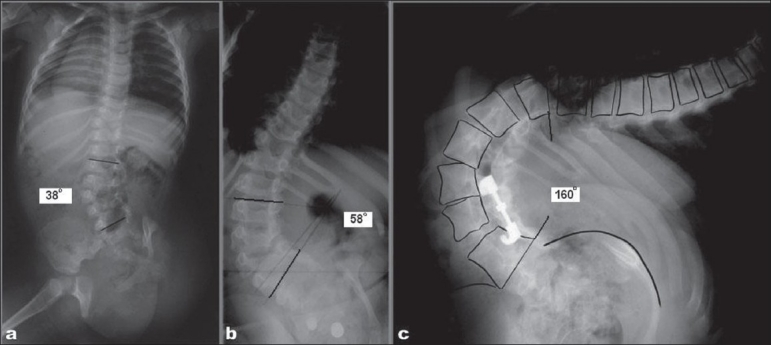
(a) One year old female with unilateral hemivertebrae at two levels (T10 and L3) and associated absence of hemipelvis and absent right kidney with a 38° left sided lumbar curve. At two years her spinal curvature increased to 48°. (b) At six years of age her curvature increased to 58°. (c) At the age of 16 years she had a 160° curvature even after hemivertebra excision and fusion

Two unilateral HV have a worse prognosis since there is absence of four growth plates on one side of the spine resulting in much higher growth imbalance. These curves progress by 3°-4° per year. All these may reach more than 70° by the end of skeletal maturity [Figure 1], therefore require prophylactic treatment to balance the spine.^26^ Block vertebrae are rare and produce a benign curve less than 20°. Miscellaneous *congenital* curves include double curves and anomalies extending throughout the spine and producing a series of small curvatures. Mixed deformities are unpredictable and need observation till skeletal maturity.

## PATIENT EVALUATION

### Clinical

A comprehensive prenatal history from the mother, including other siblings, should be recorded. Birth history of the child should include length of gestation, type of delivery, birth weight and complications. During infancy the record of developmental milestones should be noted since cognitive delay has been shown to correlate with curve progression.

The physical examination should include head to toe, especially to observe the facial features. Hemifacial microsomia (vertebral abnormalities associated with unilateral failure of formation of face and ear) is present in 2% patients with *congenital spinal deformities*.^30^ Skin must be examined for “café au lait” spots or axillary freckles and presence of neurofibromas. Hairy midline patches may suggest evidence of spinal dysraphism. The lower limbs should be examined for cavus or club feet, vertical tali and any neurological deficit. The whole spinal examination should include record of obvious deformities with truncal imbalance, abnormalities of scapula with shoulder and pelvic tilt. In the upper thoracic curves, elevation of the shoulder on the convexity of the curve, with tilting of the head into the concavity, may be seen. Structural congenital curves do not show any prominent rib hump. Unbalanced curves in the thoracolumbar and lumbar region produce a pelvic obliquity with apparent shortening of the leg on the concave side of the curve. The trunk tends to list away from the apex of the curve, and this can cause difficulty in ambulation and balance. Rib cage deformities, chest or flank asymmetry, chest excursion and anomalies need to be evaluated.

Congenital rib anomalies occur most commonly on the concavity of a *thoracic* or *thoracolumbar* congenital scoliosis that is due to a unilateral failure of vertebral segmentation, and they do not appear to have an adverse effect on curve size or rate of progression.^31^ Limitation in chest wall excursion may indicate a syndromic scoliosis and thoracic insufficiency syndrome.^32^ Curve flexibility can be assessed by placing the child in a lateral position over the knee of the examiner or by suspending the infant over the arm of the examiner. A detailed neurological examination is carried out which should include, motor, sensory and reflexes. Abdominal reflex should be part of routine tests since it's an objective finding seen in some patients with Chiari malformation.^33^

Standing or sitting photographs provide a good record of the progress of the growth of the spine and the curvature. Respiratory function tests with full spirometry work-up are recommended in all patients; especially vital capacity. For any given Cobb angle the loss in vital capacity was approximately 15% greater in congenital compared to *idiopathic scoliosis*.^34^

### Imaging

Plain radiographs remain the gold standard of imaging studies in diagnosis of congenital bony malformation, measurement of curve magnitude, progression and perhaps assessment of the growth potential of the vertebral anomaly. It is difficult to assess the radiographs because of patient's size, complexity of the deformity and superimposed bony structures. Preoperative computerized tomography (CT) helps to define the morphology of the spinal anomaly along with other associated defects. A three-dimensional reconstruction with sagittal multiplanar reformatting can be very useful in HV assessment. CT also helps to understand the chest wall deformities and lung volumes specially. in patients with thoracic wall insufficiency syndromes. Improvements in lung functions could be measured by CT following expansion thoracoplasty.^35^

All *congenital scoliosis* patients should undergo MRI scans preoperatively. Imaging of the brainstem to the sacrum is required to exclude associated conditions of the spine, the *cranio-vertebral* junction and the viscera. A T2w image through the apex of the curve and a T1w image to identify any cord abnormalities are essential.^25^

## MANAGEMENT

### Historical perspective

*Srimad Bhagwat Mahapuranam*, an ancient Indian religious literature written between 3500 BC and 1800 BC, is the oldest existing reference to axial traction for the treatment of spinal deformity. One story tells of Lord Krishna correcting the hunchback of one of his devotees, Kubja, by applying axial traction. Kubja's back was deformed at three places; probably having *scoliosis* or *kyphoscoliosis* with one primary and two secondary curves.^36^ Hippocrates (460-370 BC) was the first to invent devices based on principles of axial traction and three point correction for correction of curvatures of the spine and the management of spinal diseases. The devices used by Hippocrates for treatment of spinal deformities were the Hippocratic ladder, the Hippocratic board and the Hippocratic bench.^37^ Galen (131 AD to 201 AD), a follower of Hippocrates, used axial traction with direct pressure. Ibn Sena (980 AD to 1037 AD) in the Middle East also used similar methods. Osteopaths of Turkey also used axial traction to correct spinal deformities. But gradually mechanical methods for the correction of the spinal deformity went into disrepute due to the invariable production of paraplegia.^36^

### Goals of treatment

The primary goal of treatment of *congenital scoliosis* is to prevent the development of a severe deformity, to achieve a straight spine and preserve as much normal growth as possible. One does not need to wait until a severe deformity has developed and then attempt to perform a major and dangerous corrective procedure. For patients with a marked spinal growth imbalance, no treatment is perfect.^38^ The best result that can be achieved is spinal growth that is balanced on the convexity. In these circumstances, the optimum result is a short, relatively straight spine rather than the severely crooked spine that would have developed without treatment. To achieve optimum results in patients with congenital scoliosis one must keep in mind the following three key factors:

- Early diagnosis-If the diagnosis is made early (before the age of five years in a child), while the curvature is still small and flexible, an opportunity exists for prophylactic surgery to balance the growth of the spine.
- Anticipation-The prognosis for deterioration of *congenital scoliosis* can be anticipated based on the amount of spinal growth remaining, the type and site of the vertebral anomaly, and the degree of growth imbalance that it produces. This requires careful study of good-quality spinal radiographs and knowledge of the natural history of the condition.
- Prevention of deterioration-To prevent a severe spinal deformity is easier than to correct it.^39^ A unilateral unsegmented bar, with or without contra lateral HV, is associated with a poor prognosis and therefore requires immediate surgical treatment no matter how young the patient. Other types of *congenital scoliosis* may be observed, but one of the most common errors is the failure to recognize slow and relentless progression until it is too late for prophylactic treatment. Therefore, all patients require radiological assessment at four to six-month intervals; once progression is established, immediate treatment is necessary to prevent further deterioration.

### Non-operative

Most *congenital scoliotic curves* are nonflexible and therefore resistant to correction by bracing. Bracing can be used to prevent progression of secondary curves that develop above and below the congenital curve causing imbalance. As long as the curve remains controlled, bracing in such cases can be continued till skeletal maturity. Ideal indication for bracing is long curve involving at least eight or more vertebrae and has at least 50% flexibility.

### Surgical management

#### Principles

Congenital spinal deformities require careful planning prior to surgery. The risk of high intra-operative neurological deficit^40^ calls for a mandatory neurological monitoring. *Kyphotic* deformities are more at risk. Preoperative halo traction (in bed or in wheelchair) may be required in severe deformities which have been shown to be an effective preoperative tool.^41^ Preoperative MRI is also mandatory to rule out any intraspinal pathology. The author and colleagues use motor-evoked potential and somato-sensory evoked potential to minimize the risk for a neurological deficit.^42^ A wake-up test should also be performed which is effective in young patients.^43^ We have had successful wake-up tests in two-year-olds. Referral to a neurosurgeon is mandatory if a patient has co-existing spinal cord abnormality. Cord tether release operations could be done at the same time as HV resections or *in situ* fusions. Standard positioning on a radiolucent spinal bed with headpiece, chest bolster and iliac crest pads are pre-requisites. For younger patients, *gel* rolls are sufficient to support the torso. A standard lateral decubitus position is required for open thoracic or thoraco-abdominal procedures. Patients undergoing simultaneous anterior and posterior procedures should be placed in the lateral decubitus position (convex side up) on a flat radiolucent operating table with entire anterior and posterior fields draped out.^44^ Monitored controlled hypotension during operation is required to minimize blood loss to prevent any cord ischemia during the correction of the curve. Instrumentation in pediatric spine should be done after a preoperative CT evaluation of the pedicle anatomy. Size specific implants are available which should be used judiciously.^45^ Titanium instrumentation should be used in all patients with congenital spine deformities since it is MRI compatible. Further imaging of the spinal axis and other organs becomes easier to visualize and interpret. Studies have suggested that instrumentation is feasible and safe in smaller patients who have congenital spine deformities.^46^ The rate of union and correction of maintenance for posterior fusion is greater with instrumentation. Pedicle screw placement is safe and technically possible even in one year olds.^47^–^50^

To create spinal stability and maintain the curve correction till fusion is attained, surgeons should be prepared to use hook or screw fixation at the required levels. Although autologous iliac crest bone graft is the gold standard, it should be reserved for older children due to limitations of harvesting in the younger and smaller children. Local grafts from rib resections during anterior exposure are ideal in smaller children. In some institutions allografts (freeze dried cortico-cancellous bone chips) may be available but these are expensive.^51^ Crankshaft phenomenon is less predictable in congenital scoliosis.^52^ Therefore, one should assess the potential for growth of the spine by determining the clarity of definable discs anteriorly.

### Surgical procedures

#### In situ fusion

*In situ* fusion remains the most safe and reliable operation for congenital spine deformities. *In situ* fusion does not correct the deformity but is effective in controlling the progression of the curve. The ideal patient is one with unilateral failure of segmentation such as unilateral unsegmented bar with contra-lateral HV.^53^,^54^

*In situ* fusion should be accomplished early in life before any significant curve develops. Other ideal indication is a deformity due to a congenital failure of formation that is less than 50° in a child with significant growth potential.^55^ *In situ* fusion addresses a short segment and there is less rotation in the segment which makes a true crankshaft phenomenon unlikely. The curves due to HV at the junctional regions of the spine are more likely to lead to cosmetically disfiguring deformities and should have early *in situ* fusion. John Moe (1958) first published the critical analysis of fusion in *congenital scoliosis*.^56^

#### Convex epiphysiodesis

This procedure prevents future deformity and requires growth to obtain correction over time.^57^ The ideal anomaly is a unilateral failure of formation (fully segmented HV) without any associated deformity. This is done as an anterior and posterior fusion on the convex side of the curvature by removing lateral half of adjacent discs. The concave side retains its growth potential and allows for some correction as the child grows. Winter *et al*. (1988) suggested that this operation should be reserved for patients younger than five years of age with a progressive curve of <70° involving five segments or less and presenting with a pure *scoliosis* not involving the *cervical* spine.^58^ It is contraindicated if there is no concave growth potential as well as if the patient has any sagittal deformity.

#### Hemivertebra excision

The ideal patient is a child younger than five years of age with a fully segmented Hemivertebra (HV) at the junctional regions of the spine (*cervico-thoracic* or *thoracolumbar* or *lumbosacral*). HV can be resected by an anterior-posterior procedure or a posterior procedure only. Ruf and Harms (2002) reported success with the posterior only procedure and suggested that it is less invasive and equally effective in correcting local deformities.^59^ Suk *et al*. (2005) suggested that increased operative time in combined procedure may increase the risk of complications and therefore only posterior technique offers the advantage of single stage surgery, the ability to address the deformity at the apex and of controlled shortening across the resection gap.^60^ We believe it is a demanding procedure since this needs one to work around the cord or cauda and may make visualization difficult with increased blood loss. On the other hand, advocates of combined procedure suggest that it has greater correction ability including correction in the sagittal plane secondary to disc excision.^61^ This approach gives better visualization of the anatomy. This also allows the surgeon to apply corrective forces from an anterior position while simultaneously applying compressive instrumentation posteriorly. This also reduces the risk of pseudoarthrosis and prevents the development of crankshaft phenomenon by the removal of growth plates.

#### Single/double stage correction with instrumentation and fusion

**Figure 2:**
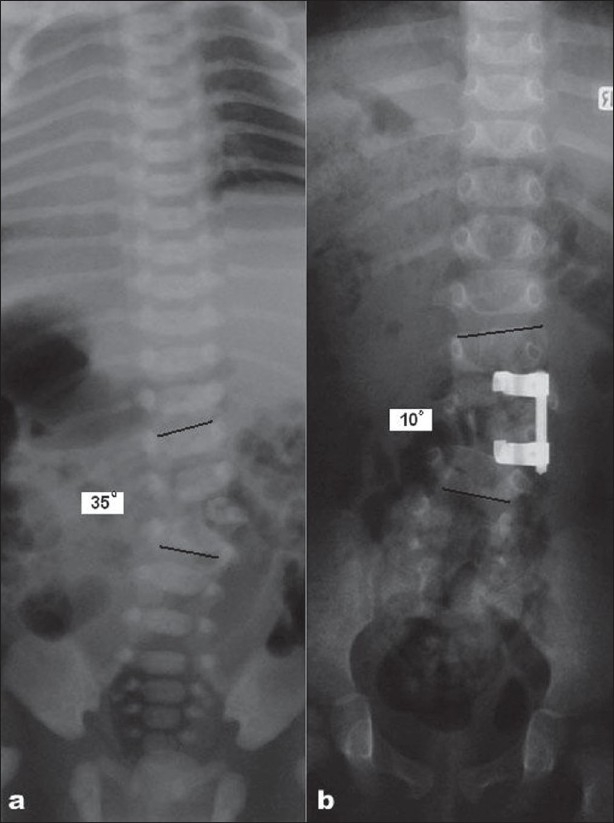
(a) A fully segmented hemivertebra at right L3 of a child of 18 months old with the curve measuring 35°. (b) She underwent posterior excision with posterior short segment fusion correcting the Cobb angle to less than 10°

The partial or complete correction of the deformity depends on the type, site and degree of curvature without compromising the neurological function. Higher *thoracic* and *cervical* curves need special precaution as overcorrection of a lower *thoracic* or *thoraco-lumbar* curve may lead to shoulder or neck obliquity. Congenital flexible curves with normal segmentation and mild to moderate truncal imbalance may be managed by single stage posterior fusion and instrumentation. One must not attempt to correct fully a large and stiff congenital curve. It is beneficial to do anterior release and posterior fusions in two stage procedure in such curves. In the earlier reports posterior fusions were fraught with pseudoarthrosis due to lack of instrumentation.^53^,^62^ Modern segmental instrumentation has improved the maintenance of correction and also the fusion rate.^46^ When trying to balance a decompensated spine in *congenital scoliosis* one applies the principle of stable vertebra. Most often we can balance the spine with posterior fusion and instrumentation [Figure 2]. One must be careful not to apply distractive forces with implants or to attempt significant corrections with instrumentation in rigid deformities.

#### Reconstructive osteotomy

The indications of osteotomy to correct the spinal balance are increasing pelvic obliquity, severe truncal decompensation, progressive deformity and evolving neurological deficit. These must be done along with anterior releases. The planning of the osteotomy is facilitated by preoperative 3D CT scans and rapid prototyping.^63^ These are challenging cases and should be done by experienced surgeons. An osteotomy may be a part of a combined approach that involves resection of the HV and instrumentation and fusion of a more extended curve. Transpedicular eggshell osteotomy has been described for treating older patients with multiple anomalies and multiplanar deformity. Screws and hooks are placed above and below the osteotomy site before doing the osteotomy close to the end plates.^64^

#### Vertebral column resection

This procedure is rarely needed and can be a salvage procedure for severe deformity that cannot be managed by other means. They have been performed safely with good results.^59^,^65^ Ideally they are indicated in *kyphotic* deformity which is causing spinal cord compression. Vertebral column resections may be done as a staged anterior-posterior procedure or posterior only resection. Combined procedures are staged because of significant bleeding and are technically demanding. Resection of the anterior body is done all the way back to the posterior longitudinal ligament. Following complete resection, abundant autograft and allograft are placed to fill the gap. The posterior resection is carried out a week later. Frequently, multiple wake-up tests are required along with good neural monitoring.

#### Growing rods

Growing rod technique was initially reported by Paul Harrington in 1962.^66^ John Moe *et al.* used the technique specifically for growing children referring to it as ‘subcutaneous rod technique’.^67^ Marchetti and Faldini described the technique of limited ‘end fusions’ to enhance the stability of implants at the anchor sites.^68^ Most of these previously described methods have used single rod construct. Luque instrumentation without fusion,^69^ referred to as the ‘Luque trolley’, has been in use since 1980s with long term follow-up studies revealing unwanted autofusion.^70^ Akbarnia (2005) first introduced the concept of dual growing rod technique for early onset scoliosis and he has been using this technique for treating children with *congenital scoliosis*.^71^

*Congenital scoliosis* is associated with short stature and diminished trunk height. Further fusions can diminish the trunk height and diminish the thoracic volume leading to thoracic insufficiency. The goal of treatment with growing rods is to provide deformity correction and simultaneously allowing for continued spinal growth. Definitive fusion and instrumentation is carried out once maximal spinal growth is achieved. The age of five years is considered critical in a child's life when the child reaches two-thirds of the normal sitting height of an adult and the *thoracic* volume reaches 30% of adult size. Growing rods rely on normally segmented areas of spine to maintain growth while instrumentation aids in curve control. Proximal and distal anchors are placed by using claw constructs and spanning submuscular rods to gain correction while allowing growth. Lengthening is done every six months. The ideal patient is younger than five years of age and has a *congenital* deformity involving a long segment of the spine in which fusion would be deleterious for the growing spine. It is nowadays preferable to use dual rod construct instead of single rod since there is increased risk of hook dislodgement and rod breakage in single rod construct. Dual growing rod technique resulted in 5.7±2.9 cm of spinal growth during a 4.37±2.4 year treatment period. There was significantly greater growth and correction achieved in those lengthened more frequently.^72^

#### Expansion thoracoplasty and vertebral expandable prosthetic titanium rib

*Congenital spinal deformities* may be associated with fused ribs which can cause progressive diminution of pulmonary function and hemithorax volume.^31^ The resultant poor thoracic and lung parenchymal maldevelopment causing limitation of pulmonary function was termed ‘thoracic insufficiency syndrome’ by Robert Campbell (2003).^32^ The first eight years of life see maximum growth of the lung, bronchial tree and alveolar cell multiplication;. 50% of thoracic volume is attained by the first 10 years of life. Early spinal fusion in patients with pre-existing thoracic insufficiency syndrome may compound the effect of poor pulmonary function in a growing child. The progressive loss of hemithorax height is believed to be responsible for this loss of function. Expansion of the hemithorax by opening wedge osteotomies into an area of congenital rib fusions or adhesions was shown to have a positive effect on pulmonary function and lung volume.^73^ The vertebral expandable prosthetic titanium rib (VEPTR) holds the expanded hemithorax in place which anchors proximally around the 2^nd^/3^rd^ ribs. Distally the VEPTR may anchor into one of the three places: around distal ribs, into the *lumbar* spine or around the ilium bone. The device can be expanded through connectors every six months. This improves the thoracic height, lung volume and lung function. The ideal indication is a patient who has a constricted hemi-thorax secondary to congenital rib fusions.^74^

## OUTCOME

Reports of combined anterior and posterior HV excision have shown good curve correction with little deterioration over time. The mean postoperative curve correction obtained in HV resections in all parts of the spine range from 59 to 67% of the initial curve with little loss of correction at least two years post-operatively. HV resection via posterior approach has been reported to correct between 23°-36° with an average total of 3.7° loss of correction at final follow-up.^59^,^75^ In a long term study for single, fully segmented HV with single stage excision via posterior approach alone accounted for 54.3% *scoliosis* correction and 67.4% *kyphosis* correction.^76^ In one study of six very young children who had sequential single stage anterior and posterior HV excision had mean postoperative correction of 67%.^77^ The results of a *lumbar* HV resection and short- segment fusion through a lateral-posterior approach reported postoperative curve correction of 60.9%.^78^ In a study of 10 patients with *thoracic* and *thoracolumbar* HV excision, the mean curve improvement was 59%.^79^ The correction achieved with *lumbosacral* HV excision is much less as compared to other regions (10°-12° of correction). Hosalkar *et al.* (2004) evaluated the efficacy of the excision of a *lumbosacral* HV followed by fixation of the normal adjacent vertebra to the ilium with screws and cables. This was found to be biomechanically stronger construct in infants and young children with soft bones.^80^

In a long term follow-up (10-52 years) study from Nottingham, 52 patients (18M: 34F), had primary growth arrest and fusion. The etiology included: hemivertebrae (n=22), unsegmented bar (n=15), unsegmented bar with contra lateral HV (n=4), wedged vertebra (n=5), hemi-metameric shift (n=2) and unclassifiable (n=4). Co-existent intra-spinal anomalies were seen in 10 (19%) and associated syndromes in 11 patients. We grouped the patients into three surgical groups.

Group I: posterior *in-situ* fusion (n=16),

Group II: anterior/posterior correction and fusion (n=32)

Group III: anterior HV excision with correction and fusion (n=4).

The results suggested the following:

1. Growth arrest and fusion performed at an early age can deteriorate over time especially if a stand-alone posterior *in-situ* fusion is performed.
2. The timing of definitive surgery was influenced by type, site and number of HV and presence of unsegmented bar.
3. Early definitive HV excision/fusion is recommended, especially for junctional HV in preventing long-term rigid deformities.^81^

Winter and Lonstein (2009) reported a 51 year follow-up study of 23 patients, eight patients of them with *congenital* spinal deformity. They reported that early spine fusion for deformity produced far better results than delayed fusion. A solid fusion at the end of growth remained unchanged.^82^

## FUTURE

Laboratory as well as clinical studies suggests the need for VEPTR in patients with severe congenital scoliosis. Hell *et al.* (2005) reported the clinico-radiological improvement in spinal deformity treated with VEPTR.^83^ As we know more about the technology and improve our growing rod technique we shall be able to minimize the number of operations in a child required to maintain the growth of the spine. Advances in genetic research may help in future to detect the spinal defect early *in-utero* and modulate the treatment with gene therapy.

## CONCLUSION

Congenital scoliosis is fraught with dilemmas in the minds of treating surgeons. In the last 50 years we have known more about the natural history of different types of congenital scoliosis and their behavior in the growing spine. One has to be astute in applying the correct principles for a successful outcome. A thorough preoperative assessment is essential and the correct surgical procedure depends on the anomaly itself and the degree of deformity. Early diagnosis and surgical treatment is the hallmark of successful outcome. Mild to moderate deformities can be managed successfully with fusion and instrumentation. HV excision in junctional areas of spine is necessary to correct the deformity. Posterior approach alone or in combination with anterior approach depends on the patient, the type of anomaly, the deformity and the surgeon. Occasionally, osteotomies may be necessary to balance the spine. Curvature in young children with long normally segmented areas of the spine may be managed by growing rods. Patients with associated thoracic insufficiency may be best treated by VEPTR.
